# The Morphology and Intrinsic Excitability of Developing Mouse Retinal Ganglion Cells

**DOI:** 10.1371/journal.pone.0021777

**Published:** 2011-07-13

**Authors:** Juan Qu, Karen L. Myhr

**Affiliations:** 1 Department of Biological Sciences, Wayne State University, Detroit, Michigan, United States of America; 2 Department of Anatomy and Cell Biology, Wayne State University, Detroit, Michigan, United States of America; Tokyo Medical and Dental University, Japan

## Abstract

The retinal ganglion cells (RGCs) have diverse morphology and physiology. Although some studies show that correlations between morphological properties and physiological properties exist in cat RGCs, these properties are much less distinct and their correlations are unknown in mouse RGCs. In this study, using three-dimensional digital neuron reconstruction, we systematically analyzed twelve morphological parameters of mouse RGCs as they developed in the first four postnatal weeks. The development of these parameters fell into three different patterns and suggested that contact from bipolar cells and eye opening might play important roles in RGC morphological development. Although there has been a general impression that the morphological parameters are not independent, such as RGCs with larger dendritic fields usually have longer but sparser dendrites, there was not systematic study and statistical analysis proving it. We used Pearson's correlation coefficients to determine the relationship among these morphological parameters and demonstrated that many morphological parameters showed high statistical correlation. In the same cells we also measured seven physiological parameters using whole-cell patch-clamp recording, focusing on intrinsic excitability. We previously reported the increase in intrinsic excitability in mouse RGCs during early postnatal development. Here we showed that strong correlations also existed among many physiological parameters that measure the intrinsic excitability. However, Pearson's correlation coefficient revealed very limited correlation across morphological and physiological parameters. In addition, principle component analysis failed to separate RGCs into clusters using combined morphological and physiological parameters. Therefore, despite strong correlations within the morphological parameters and within the physiological parameters, postnatal mouse RGCs had only limited correlation between morphology and physiology. This may be due to developmental immaturity, or to selection of parameters.

## Introduction

The retina has long been a model system in which to study neuronal morphology and physiology, i.e. neuron structure and function [1]–[7]. Early physiological categorizations of retinal ganglion cells (RGCs) were based on light responses, such as ON versus OFF responses and large versus small receptive fields [6], [8]–[10], but other physiological parameters also affect function and have been used to categorize RGCs [4], [11]–[14]. In general, cat RGCs can be divided into three physiological types (X, Y and W) based on different light responses. These three different physiological types correspond to three morphological classes (beta, alpha and gamma) based on soma size, dendritic field size and dendritic branching pattern [2], [6], [15]–[18]. Although morphologically identified cat alpha and beta RGCs have equivalent intrinsic temporal properties [19], their intrinsic physiological properties (e.g., resting potentials, spike widths and maximum spike frequencies) differ significantly [4]. However, this high level of specificity between morphology and physiology in cat RGCs is partially lost in rat RGCs [20].

The mouse retina has become an increasingly valuable model for vision research allowing the genetic exploration of the relationship between morphology and intrinsic excitability in RGCs. Mouse RGCs can be divided into ON, OFF and multistratified RGCs. Their dendrites stratify in ON and/or OFF sublaminas in the inner plexiform layer (IPL) and they respond to the onset and/or the offset of the light stimulation. Mouse RGCs increase in size and gain different morphology and physiology with maturation. In mouse, the adult-like morphology becomes apparent after P8 [21], [22] with about a dozen morphological subtypes [23]–[27]. The intrinsic excitability increases with maturation during the first three postnatal weeks and a greater proportion of RGCs gain the ability to fire action potentials repetitively [28]. Although in adult mouse ON and OFF RGCs express different ion channels and display some different firing mechanisms [29], [30], their intrinsic excitability is not different during the first three postnatal weeks [28]. However, our previous ON/OFF morphological classification only took into account the depth of stratification of the dendrites in the IPL, not other parameters such as dendritic field sizes which would correspond to receptive field sizes. Here, we comprehensively characterized the development of the morphological properties in mouse RGCs in the first four postnatal weeks, when the morphological subtypes become apparent and the retinocollicular projection is formed and refined. We determined the correlation among the morphological parameters, the correlation among the physiological parameters that quantify intrinsic excitability, and the correlation between the morphological parameters and the physiological parameters through postnatal development in order to understand how diversity develops. This is an important foundation to understanding the relationships between morphology and physiology in the adult and how the diversity of neuronal form and function is regulated. To our knowledge, this is the first study with simultaneous recording of morphology and physiology spanning a broad spectrum of mouse RGC subtypes during the critical postnatal development period.

## Materials and Methods

### Tissue preparation

C57Bl/6 mice were used in this study. All procedures were carried out with the approval of the Institutional Animal Care and Use Committee of Wayne State University (protocol #: A 05-09-09). Tissue preparation, electrical recordings and measurements were the same as described previously [28]. Briefly, P4-24 C57Bl/6 mice were anesthetized and euthanized. The eyes were enucleated, and the retinas were isolated, mounted on non-fluorescent filter paper (HABG01300, Fisher Scientific, Pittsburg, PA) and kept in oxygenated artificial cerebrospinal fluid (ACSF; in mM: NaCl, 119.0; KCl, 2.5; MgCl_2_, 1.3; CaCl_2_, 2.5; NaH_2_PO_4_, 1.0; Glucose, 11.0; HEPES, 20.0 adjusted to pH 7.4 with NaOH).

### Electrical recordings and physiological analysis

Current-clamp recordings were made using MultiClamp 700A (Axon Instruments, Inc., Union City, CA) in whole-cell configuration. Intracellular solutions contained (in mM): KCl, 120; NaHEPES, 10; or KMeSO_4_, 100; KCl, 20; NaCl, 5; EGTA, 5; HEPES, 10; or KGluconate, 120; NaCl, 12; MgCl_2_, 2; CaCl_2_, 1; HEPES, 10; EGTA, 1.5; ATP, 2. The pH of intracellular solutions was adjusted to 7.3 with 5 M KOH. Membrane resistance (Rm), membrane capacitance (Cm), resting membrane potential (Vm), action potential threshold (APT), action potential width (AP Width) and maximal instantaneous firing rate were measured as described previously [28]. The same dataset was analyzed further in this study, except RGCs with inadequate image quality and RGCs with large dendritic fields that extended beyond the field of view (20X objective, 430 by 328 micrometers; 40X objective, 215 by 164 micrometers) were excluded. Additional RGCs were recorded in this study to increase the sample size. The composition of the RGCs at different ages from the previous dataset and newly recorded is listed in Table S1.

### Morphological measurements

The intracellular solution contained Alexa 647 hydrazide triethylammonium salt (A20502, Invitrogen, Carlsbad, CA) or Lucifer Yellow (L0144, Sigma-Aldrich, St. Louis, MO), which filled the dendrites for the visualization of the neuronal morphology. Following each recording, a stack of pictures were taken every 1 micrometer using a 20X or 40X objective (0.5 and 0.8 NA, respectively) on an Olympus BX51-WI with a Retiga Exi camera (QImaging, Surrey, BC, Canada) and MetaMorph software (Molecular Devices, Sunnyvale, CA).

**Figure 1:**
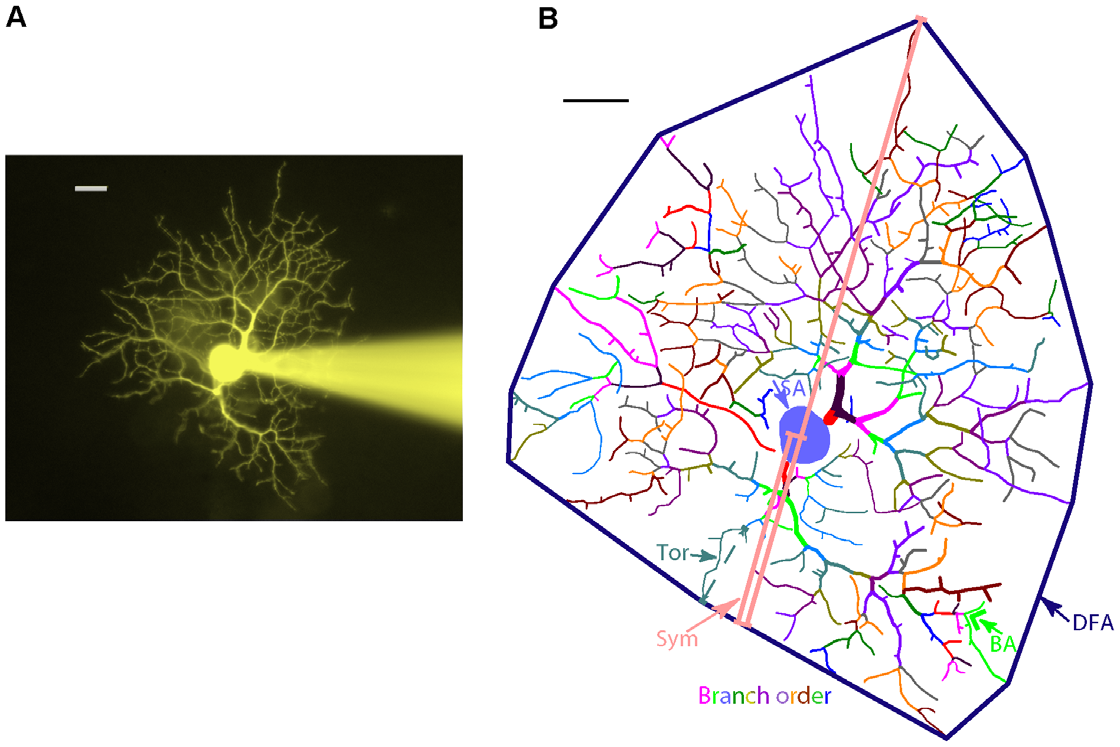
Measurement of morphological parameters. (A) Image of a recorded RGC. Fluorescence dye in the recording pipette diffused into the RGC and revealed its morphology. (B) The digital reconstruction of the RGC shown in A. A stack of images were imported into Neurolucida and the RGC was digitally reconstructed in three dimensions. The measurements of soma area (SA), dendritic field area (DFA), branch order (different color of the branches), branch angle (BA), tortuosity (Tor) and symmetry (Sym) are indicated. Scale bars represent 20 µm.

**Figure 2:**
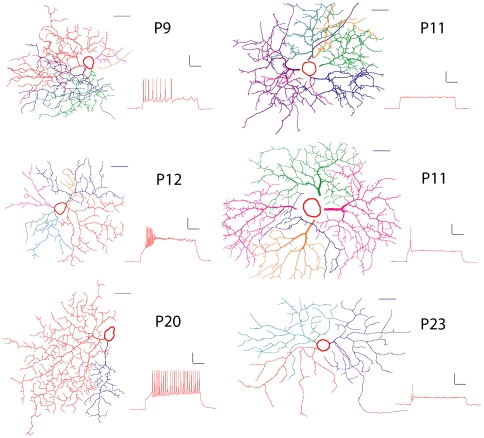
Samples of digitized RGCs with their different firing patterns. RGCs vary greatly in their morphology and fire action potentials differently in response to injected current. Scale bars represent 20 µm (horizontal lines), 20 mV (vertical component of physiology scale bars) or 200 ms (horizontal component of physiology scale bars).

The dendritic morphology was digitally reconstructed for quantification. Image stacks were imported into Neurolucida (Microbrightfield, Inc., Williston, VT) and the width and position of each segment of dendrite and branch point was digitized (Fig. 1). Twelve morphological parameters were measured for each neuron. Eleven of these parameters were the same as the parameters defined by Coombs and colleagues [21], [24]. Soma area was the area of the horizontal section that went through the center of the RGC soma. Dendritic field area was the area of the region defined by connecting the outermost tips of the dendrites. Total dendrite length was the total length of all the dendrites. Number of dendritic branches was the total number of all the branches in all the dendrites. Branch order was the largest number of times that a dendrite branched for an RGC. Mean internal branch length and mean terminal branch length were the average length of all the internal branches or terminal branches, respectively. Branch angle was the average angle in three dimensions formed by two dendrites leaving the branch point. Dendrite diameter was the average diameter of all the primary, secondary and tertiary branches. Tortuosity was the ratio of the length of each branch divided by the straight distance between the two ends of this branch. Symmetry was the ratio of the distance from the soma to the closest edge of the dendritic field divided by the radius of the dendritic field. The twelfth parameter was dendrite density, which was found important for RGC morphology by Kong and colleagues [26]. Dendrite density was defined as total dendrite length divided by dendritic field area. Each RGC was identified as an ON, OFF or multistratified RGCs according to dendritic stratification as described previously [28]. Samples of digitized RGCs and their firing patterns are shown in Figure 2.

### Statistical analyses

Statistical analyses were performed using Excel and SPSS version 16 (SPSS Inc., Chicago, IL). We used Pearson's correlation coefficient to assess the relationships among and across morphological and physiological parameters. Pearson's correlation coefficients indicate the strength and direction of a linear relationship between two random variables. A correlation coefficient of 1 indicates the two parameters are completely correlated and a correlation coefficient of 0 indicates the two parameters are completely independent. Positive values mean the two parameters are directly related and negative values mean the two parameters are inversely related.

We used principal component analysis (PCA) to extract independent factors out of the original parameters without the bias of presumptive selection and weighting. We applied PCA to twelve morphological parameters and seven physiological parameters.

## Results

### The development of morphology in mouse RGCs

**Figure 3:**
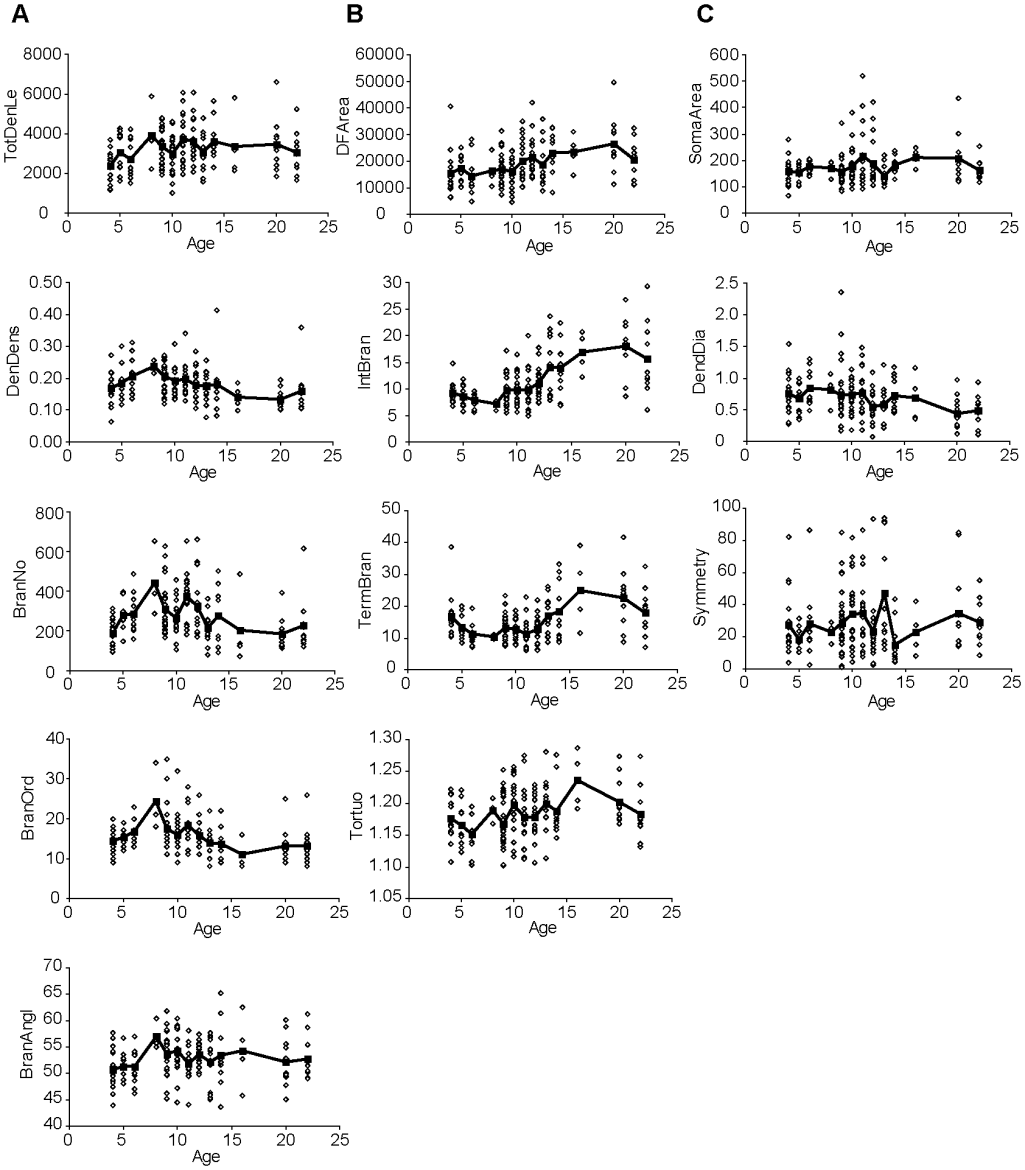
Developmental patterns of morphological parameters. (A) The morphological parameters in the first category peaked at P8. Total dendritic length, dendrite density, number of dendritic branches, branch order and branch angle increased after P4, reached the peak at P8, and then decreased after P8. (B) The morphological parameters in the second category peaked at around P16–20. Dendritic field area, mean internal branch length, mean terminal branch length and tortuosity decreased or didn't change much after P4, reached a minimum at around P6–8, and then increased and reached a maximum at around P16–20, and decreased again at P21–24. (C) The morphological parameters in the third category did not change much during postnatal development. Soma area, dendrite diameter and symmetry did not change much from P4 to P24. Open diamonds represent measurements of individual RGCs, filled squares are the average value at each postnatal age, and solid lines connect the average values.

The morphology of mouse RGCs changes as the RGCs mature. To determine the developmental progression of RGC morphology, 175 RGCs from mice aged between P4 and P24 were patch-filled with fluorescent dye, imaged and digitally reconstructed. Twelve morphological parameters were measured and their developmental patterns fell into three categories defined by the patterns of increases and decreases through development. The first category includes parameters that increased after P4, reached a peak at P8, and then were stable or decreased after P8 (Fig. 3A). The parameters with this pattern included the total dendrite length, dendritic density, number of dendritic branches, branch order, and branch angle. The second category included parameters that did not change much or decreased after P4, reached a minimum at around P6–8, and then increased and reached a maximum at around P16–20, and decreased again at P21–24 (Fig. 3B). This category included dendritic field area, mean internal branch length, mean terminal branch length and tortuosity. The parameters in the third category were relatively constant from P4 to P24 (Fig. 3C). This group included soma area, dendrite diameter and symmetry.

The two major turning points for mouse RGC morphological development were P8 and P16–20, which coincide with the timing of two major developmental events in the retina. P8 is when the bipolar cells start to contact RGCs, and P16–20 is a few days after eyes open. Therefore, the development of the parameters in the first category that peaked at P8 might affect or be strongly affected by bipolar cell contact but not the visual input; the development of the parameters in the second category that decreased between P4 and P8 then increased again after P8 might affect or be affected by both the bipolar cell contact and visual input; and the parameters in the third category that were stable through development might be independent of bipolar cell contact and visual input.

Based on these two critical developmental time points, the RGCs were divided into three age groups: P4–6, P9–14 and P20–24. We compared the morphological parameters across these age groups. The average values of the twelve morphological parameters are listed in Table 1. Six of the twelve parameters (dendrite density, number of branches, branch order, internal branch length, terminal branch length and branch angle) were significantly different among different age groups (ANOVA, p<0.05), indicating refinement over postnatal development; other parameters (soma area, dendrite diameter and symmetry) were not different.

**Table 1:** Comparison of morphological parameters across RGCs at different developmental ages.

	p (ANOVA)	P4–6		P9–14		P20–24	
**Category 1**							
Total Dendrite Length (µm)	0.155	2580	a	3164	b	3026	a,b
Dendrite Density (µm^−1^)	0.003	0.178	a	0.195	a	0.135	b
Number of Branches	0.012	242	a	284	b	183	a
Branch Order	0.022	15.8	a,b	16.0	a	12.1	b
Branch Angle (°)	0.024	50.3	a	53.3	b	51.6	a,b
**Category 2**							
Dendritic Field Area (µm^2^)	0.055	15550	a	17589	a	22960	b
Internal Branch Length (µm)	0.000	8.65	a	10.7	b	17.6	c
Terminal Branch Length (µm)	0.002	14.3	a	13.5	a	20.7	b
Tortuosity	0.145	1.16	a	1.18	a,b	1.19	b
**Category 3**							
Soma Area (µm^2^)	0.662	148	a	165	a	160	a
Dendrite Diameter (µm)	0.301	0.696	a	0.689	a	0.511	b
Symmetry	0.729	26.1	a	31.3	a	30.3	a

The parameters were grouped into three categories as shown in Figure 3. The numbers in the left column for each age were the average values of the parameters, while the letters in the right column indicated whether these values were significantly different among three ages. The values with the same letter were not significantly different from each other (p>0.05), while the values with different letters were significantly different (p<0.05).

### The correlation among morphological parameters

We used Pearson's correlation coefficient to evaluate the relationship among the twelve morphological parameters. As described above, the morphology changed as RGCs matured. Therefore, we first investigated correlations in a small age range, P9–14, to reduce the effect of developmental variation. This range was chosen because it is the period between the two major time points for RGC morphological development (Fig. 3), and the majority of the data were in this range (Table S1).

**Figure 4:**
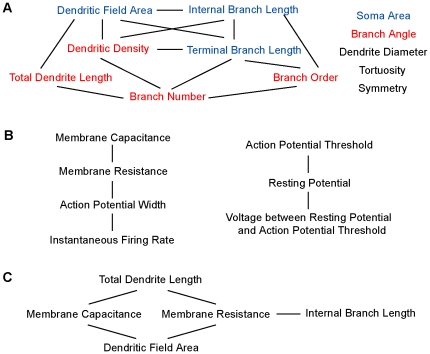
Relationship among morphological and physiological parameters. Highly correlated parameters were connected by the lines. Unlinked parameters are relatively independent from each other. Please refer to Table 2, 3 and 4 for the degrees of correlation. There were strong correlations among morphological parameters (A), among physiological parameters (B), and between a few morphological and physiological parameters (C). In (A), colors indicate parameters with different developmental patterns, i.e. peaking at P8 (red), peaking between P16–20 (blue), and stable through development (black).

Many of the morphological parameters were not independent of one another (Table 2). The mean internal branch length and the mean terminal branch length had the highest correlation coefficient of 0.848 (p<0.001), which means the RGCs that had longer internal branches also had longer terminal branches. The RGCs with larger dendritic fields had longer total dendrite lengths and longer internal and terminal branches, as would be expected for large dendritic fields, but also lower dendritic densities, which is not as directly linked to dendritic field size. This matches the impression that large RGCs had longer but sparser dendrites compared to the small compact RGCs with dense dendrites. The RGCs that had higher dendritic densities had more, but shorter, branches, and greater branch angles, which led to the more densely covered dendritic fields. The RGCs that had more branches had longer total dendritic lengths, but their dendrites branched more and the branches were shorter. The more times the dendrites branched, the shorter the branches were, which is consistent with the finding that the RGCs with smaller dendritic fields had denser dendrites. The RGCs that had shorter internal branches had greater branch angle. Soma area, dendrite diameter, tortuosity and symmetry were relatively independent parameters. Figure 4A graphically represents the relationship network among these twelve morphological parameters.

**Table 2:** Parametric Pearson correlation among morphological parameters at P9–14.

PearsonCorrelationCoefficients	Soma Area	Dendritic Field Area	Total Dendrite Length	Dendrite Density	Number of Branches	Branch Order	Internal Branch Length	Terminal Branch Length	Branch Angle	Dendrite Diameter	Tortuosity	Symmetry
Soma Area	1.000	.181	.187	−.038	.198*	.068	.012	−.051	−.039	.210*	−.095	−.168
Dendritic Field Area		1.000	.687**	−.585**	.018	−.241*	.676**	.562**	−.473**	.197*	−.164	−.138
Total Dendrite Length			1.000	.114	.567**	.094	.273**	.162	−.146	.105	.064	−.130
Dendrite Density				1.000	.542**	.371**	−.558**	−.499**	.528**	−.144	.309**	.071
Number of Branches					1.000	.660**	−.515**	−.576**	.261**	.138	.036	−.096
Branch Order						1.000	−.647**	−.572**	.254**	.183	.054	.101
Internal Branch Length							1.000	.848**	−.510**	−.025	−.014	.150
Terminal Branch Length								1.000	−.424**	.084	−.015	−.034
Branch Angle									1.000	−.198*	.384**	−.194*
Dendrite Diameter										1.000	−.145	−.021
Tortuosity											1.000	.097
Symmetry												1.000

*Correlation is significant at the 0.05 level (2-tailed).

**Correlation is significant at the 0.01 level (2-tailed).

Because the morphological and physiological parameters varied in dimension and scale, nonparametric correlation coefficients among and across morphological and physiological parameters were also analyzed. This was done by ranking the raw data from the lowest to the highest for each parameter and then evaluating the Pearson's correlation coefficient of the rankings among the parameters. The nonparametric correlation coefficient for each parameter pair was very similar to the correlation coefficients of the raw data described above (data not shown). When all the RGCs aged from P4 to P24 were analyzed as a group for both the parametric correlation and the nonparametric correlation, the Pearson's correlation coefficient for each parameter pair was very similar to the value at P9–14 (data not shown).

### The correlation among physiological parameters

We previously reported the development of RGC intrinsic excitability in early postnatal mouse [28]. As the RGCs mature, they become more excitable and gain the ability to fire sharper and faster action potentials repetitively. Here, using parametric and nonparametric Pearson's correlation coefficient we analyzed the relationships among seven physiological parameters that measure the intrinsic excitability. Some of these physiological parameters were significantly correlated. Table 3 shows the parametric Pearson's correlation coefficients of physiological parameters of P9–14 RGCs, which is graphically presented in Figure 4B. The RGCs with higher membrane capacitance had lower membrane resistance. The action potential threshold was directly related to the resting membrane potential. This is consistent with our previous results [28] that both the action potential threshold and the resting membrane potential hyperpolarized during development but the distance between them did not change. The difference between the action potential threshold and the resting potential was inversely related to the resting potential. The action potential width was inversely related to the maximal instantaneous firing rate, which indicated the RGCs that had sharper action potentials also fired faster. All these correlations were highly significant (p<0.001). The nonparametric correlation for P9–14 RGCs and the parametric and nonparametric correlation for P4–24 RGCs had similar results (data not shown).

**Table 3:** Parametric Pearson correlation among physiological parameters at P9–14.

Pearson Correlation Coefficients	Cm	Rm	Vm	APT	APT-Vm	AP Width	Firing Rate
Cm	1.000	−.592**	−.123	−.239*	.052	−.266*	.086
Rm		1.000	.033	.202	.045	.491**	−.254**
Vm			1.000	.511**	−.730**	.275**	−.149
APT				1.000	.215*	.160	−.248*
APT-Vm					1.000	−.186	−.069
AP Width						1.000	−.595**
Firing Rate							1.000

*Correlation is significant at the 0.05 level (2-tailed).

**Correlation is significant at the 0.01 level (2-tailed).

### The correlation across morphological and physiological parameters

The correlation across morphological parameters and physiological parameters were also analyzed. Despite the common impression that different RGC morphological subtypes carry different physiological functions, the correlation was very low between the morphological and physiological parameters. Table 4 shows the parametric Pearson's Correlations across morphological and physiological parameters for P9–14 RGCs, which is represented graphically in Figure 4C. The only high correlations (Pearson's correlation coefficient >0.5 or Pearson's correlation coefficient <−0.5, p<0.001) were that RGCs with larger dendritic fields and longer total dendrite length had higher membrane capacitance and RGCs with longer total dendrite length had lower membrane resistance. The nonparametric correlation for P9–14 RGCs and the parametric and nonparametric correlations for P4–24 RGCs had similar results (data not shown).

**Table 4:** Parametric Pearson correlation across morphological and physiological parameters at P9–14.

Pearson Correlation Coefficients	Soma Area	Dendritic Field Area	Total Dendrite Length	Dendrite Density	Number of Branches	Branch Order	Internal Branch Length	Terminal Branch Length	Branch Angle	Dendrite Diameter	Tortuosity	Symmetry
Cm	.371**	.652**	.737**	−.110	.338**	.000	.337**	.213*	−.305**	.210*	−.165	−.131
Rm	−.199*	−.473**	−.531**	.089	−.164	.174	−.372**	−.239*	.179	−.076	−.091	.093
Vm	−.035	−.020	−.097	−.033	−.133	−.140	−.006	.059	.018	−.009	−.084	−.073
APT	−.222*	−.126	−.300**	−.057	−.280**	−.076	.007	.036	.005	−.191	−.144	.229*
APT-Vm	.149	−.022	−.038	−.021	.027	.049	.001	−.118	.074	−.114	.013	.119
AP Width	.090	−.225*	−.284**	.010	−.078	.168	−.245*	−.074	−.042	.067	−.124	−.019
Firing Rate	−.153	.050	.071	−.010	−.009	−.106	.059	−.016	.069	−.043	.044	−.063

*Correlation is significant at the 0.05 level (2-tailed).

**Correlation is significant at the 0.01 level (2-tailed).

The measured morphological parameters of ON, OFF and multistratified RGCs were not different, except the multistratified RGCs had longer total dendrite length and higher dendrite density (Table 5). This is easily understandable because the multistratified RGCs have dendrites in both the ON and OFF sublaminas, while ON and OFF RGCs only stratify in one sublamina.

**Table 5:** Comparison of morphological parameters across ON, OFF and multistratified RGCs.

	P (ANOVA)	ON		OFF		Multi	
**Category 1**							
Total Dendrite Length (µm)	0.051	2706	a	2691	a	3487	b
Dendrite Density (µm^−1^)	0.143	0.157	a	0.166	a	0.197	b
Number of Branches	0.378	223	a	230	a	275	a
Branch Order	0.469	14.8	a	14.4	a	15.3	a
Branch Angle (°)	0.714	51.9	a	51.6	a	51.9	a
**Category 2**							
Dendritic Field Area (µm^2^)	0.759	18918	a	17020	a	19570	a
Internal Branch Length (µm)	0.014	11.2	a	11.3	a	13.9	a
Terminal Branch Length (µm)	0.541	16.3	a	14.4	a	16.9	a
Tortuosity	0.927	1.17	a	1.18	a	1.18	a
**Category 3**							
Soma Area (µm^2^)	0.989	152	a	159	a	163	a
Dendrite Diameter (µm)	0.636	0.631	a	0.592	a	0.704	a
Symmetry	0.805	26.7	a	32.8	a	28.4	a

The parameters were grouped into three categories as shown in Figure 3. The numbers in the left column for each RGC type are the average values of the parameters, while the letters in the right column indicate whether these values were significantly different among three RGC types. Within each parameter, the values with the same letter were not significantly different from each other (p>0.05), while the values with different letters were significantly different from each other (p<0.05).

We also compared the morphological parameters among the RGCs with different firing patterns (Table 6). The firing patterns were defined as in our previous report [28]. Due to the scarcity of RGCs with the sustained firing pattern, sustained and adapting firing RGCs were combined as one group because they were both able to fire action potentials to the end of the stimulation. The other two groups were phasic and single-firing RGCs. Phasic RGCs ceased firing before the end of the stimulation and single-firing RGCs only fired one action potential regardless of the level of stimulation. Compared to the other RGCs, single-firing RGCs had smaller dendritic field areas, shorter total dendrite lengths, fewer branches and shorter internal branch lengths. All of these indicated the single-firing RGCs were smaller in size and, perhaps, less mature. This is consistent with the intrinsic physiology, which also showed the single-firing RGCs had lower membrane capacitance (Cm) and higher membrane resistance (Rm) [28].

**Table 6:** Comparison of morphological parameters across RGCs with different firing patterns.

	P (ANOVA)	Sustained & Adapting		Phasic		Single	
**Category 1**							
Total Dendrite Length (µm)	0.064	3209	a	3042	a	2440	b
Dendrite Density (µm^−1^)	0.848	0.170	a	0.169	a	0.177	a
Number of Branches	0.097	258	a	251	a	207	b
Branch Order	0.747	14.4	a	14.8	a	15.4	a
Branch Angle (°)	0.697	51.8	a	52.2	a	51.3	a
**Category 2**							
Dendritic Field Area (µm^2^)	0.389	20314	a	19230	a	15250	b
Internal Branch Length (µm)	0.736	12.2	a	13.1	a	10.6	b
Terminal Branch Length (µm)	0.818	16.5	a	16.0	a	14.9	a
Tortuosity	0.851	1.18	a	1.18	a	1.17	a
**Category 3**							
Soma Area (µm^2^)	0.194	175	a	142	a	154	a
Dendrite Diameter (µm)	0.986	0.647	a	0.600	a	0.674	a
Symmetry	0.859	30.7	a	26.2	a	30.9	a

The parameters were grouped into three categories as shown in Figure 3. The numbers in the left column for each firing type were the average values of the parameters, while the letters in the right column indicated whether these values were significantly different among three firing types. The values with the same letter were not significantly different from each other (p>0.05), while the values with different letters were significantly different (p<0.05).

**Figure 5:**
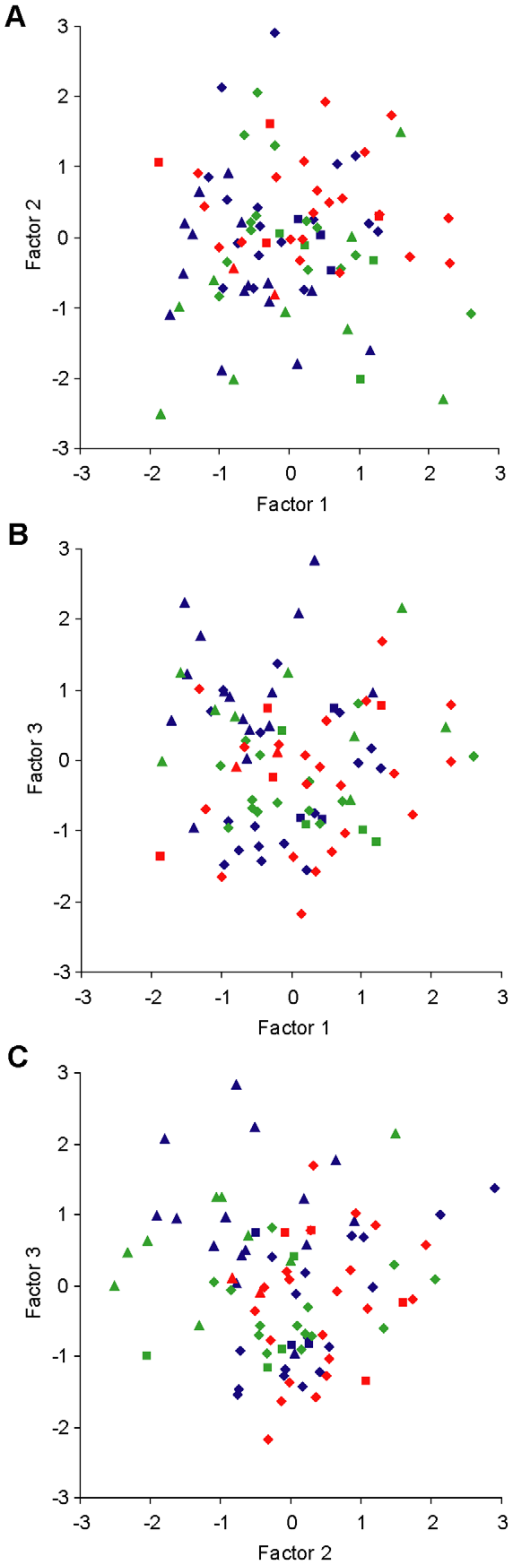
RGCs did not form isolated clusters in the plots of independent factors. Six independent factors were extracted by PCA. Factor scores 1, 2 and 3 were plotted against each other; 1 vs. 2 in A, 1 vs. 3 in B and 2 vs. 3 in C. These three factors contained 56% of the original information and have the best chance to separate RGCs. Looking at all the data points shows they did not form isolated clusters. Dividing the data into morphological types did not reveal clusters within those subsets; ON (blue) OFF (green) multistratified (red). In addition, dividing the data into firing types did not reveal clusters; sustained and adapting (diamonds), phasic (squares), single-firing (triangles).

PCA was applied to extract independent factors from the twelve morphological parameters and seven physiological parameters. These independent factors were fewer in number than the original parameters, maintained the majority of the information contained in the original parameters but removed the redundancy. At P9–14, six independent factors were extracted and they contained 78% of the variance (Table S2). Each factor was a combination of all the original parameters. The factor loading scores indicate the contribution of each parameter (Table S3). When plotted in two-dimensional space, the RGCs were randomly scattered and did not form any isolated clusters in any of the plots. Figure 5 shows the plots of the first three principal components, which explained 56% of the variance and were most likely to show clusters, if any existed. Each RGC was identified as an ON, OFF or multistratified RGCs according to dendritic stratification. When the factors were plotted to look for clusters of these identified groups, the ON, OFF and multistratified RGCs were randomly mixed together without separation (Fig. 5). Similarly, RGCs with different firing patterns did not form isolated clusters. Although RGCs that only fired single action potentials were relatively more abundant in certain regions in the distributions, they overlapped considerably with the RGCs that fired action potentials repetitively (Fig. 5). A PCA analysis of the entire age range (P4–24) was also performed. Seven principal components were extracted explaining 80% of the variance. As with the P9–14 RGCs, these RGCs from P4–24 did not form clusters in the two-dimensional plots of the principal components, neither did RGCs that stratified differently or that had different firing patterns (data not shown). Therefore, postnatal mouse RGCs did not form clusters, morphologically or physiologically, using PCA analysis.

## Discussion

We previously described the development of intrinsic excitability in mouse RGCs and that intrinsic excitability was similar among mouse ON, OFF and multistratified RGCs during the first three postnatal weeks [28]. In this study, the development of morphological properties in mouse RGCs was comprehensively characterized during the same developmental period. We also examined the correlation among and across the morphological and physiological parameters. These data lay the foundation to understand the regulation of the morphology and physiology of RGCs. Development of diversity provides a model for regulation of morphology and physiology because neighboring RGCs develop different functional roles due to the extent and type of dendritic growth, their composition of voltage-gated ion channels leading to intrinsic excitability and their synaptic connectivity. This work measured the postnatal development of mouse RGC morphology and intrinsic excitability to address the question of to what extent are morphology and physiology regulated together during postnatal development.

### The development of morphology in mouse RGCs

In this study, developing mouse RGCs were digitally reconstructed and twelve morphological parameters were measured for each RGC. The development of eleven of these twelve morphological parameters, except the dendritic density, was investigated by Coombs and colleagues [21]. The soma size and dendritic field size of mouse RGCs were also investigated by Diao and colleagues at P3, P8 and P13 [22]. The data for soma area, branch order, number of dendritic branches, total dendrite length, tortuosity and symmetry had similar values between this study and Coombs' study. Dendritic field area, mean internal branch length and mean terminal branch length were smaller in this study compared to Coombs' study. Dendritic field size in this study was also smaller than in Diao's study. This could be explained by the elimination of the very large RGCs in this study. Those RGCs had dendrites extending beyond the field of view and could not be reconstructed appropriately. Dendrite diameter was smaller and branch angle was larger in this study compared to Coombs' study. There was no apparent explanation for these variations.

The morphological parameters were divided into three categories of developmental patterns. In the first category, total dendrite length, dendritic density, number of dendritic branches, branch order, and branch angle peaked around P8 (Fig. 3A). This is when bipolar cells are making contact with RGCs, suggesting that contact by bipolar cells or correlated events stabilized dendritic length and branching. In the second category, dendritic field area, mean internal branch length, mean terminal branch length and tortuosity peaked around P16–20 just after eye-opening (Fig. 3B). The coincidence with eye-opening suggests that the onset of form vision attenuates the expansion of dendritic field areas by lengthening of segments of dendrites. In the third category, soma area, dendrite diameter and symmetry were relatively stable from P4 to P24 (Fig. 3C). This suggests that the symmetry and soma size, which are associated with specific RGC types, are established early and are not affected by contact from bipolar cells or the onset of form vision.

### The correlation of the morphological and physiological parameters

In postnatal mouse RGCs, many morphological parameters were correlated to one another, especially parameters that describe the length of dendrites and the size of dendritic fields. Some physiological parameters were also correlated, such as the action potential width and the maximal instantaneous firing rate. However, there was only limited correlation across the morphological parameters and the physiological parameters (Fig. 4).

Whether a correlation between morphology and physiology exists in RGCs has long been debated in the field. There is evidence supporting both sides. In cat RGCs, the three morphological classes – alpha (large soma, large dendritic field), beta (medium soma, small dendritic field) and gamma (small soma, large dendritic field) - are correlated with the three physiological types Y (transient responses to light stimulus), X (sustained responses to light stimulus) and W (weak sustained responses to light stimulus) [2], [6], [15]–[18], [31]. But X and Y RGCs are less distinct in mouse [32]–[34]. Some intrinsic physiological properties are significantly different among ten morphologically identified RGC subtypes in cat. For example, alpha and beta RGCs have different resting potentials, spike widths and maximum spike frequencies [4], but the intrinsic temporal properties of alpha and beta cat RGCs are equivalent [19]. There are also unique intrinsic properties such as rebound firing that are associated to specific RGC subtypes [4], [30]. The firing patterns are different between ON and OFF RGCs in developing ferret [35], [36], but not in developing mouse [28]. However, adult mouse ON and OFF RGCs do acquire some different ion channel populations and different firing mechanisms [29], [30] at ages when the synaptic drive to ON and OFF RGCs differs due to visual drive, and this extended visual experience may be required for differences in intrinsic physiology to develop. Differences in the conclusions arise depending on which parameters are measured, at which developmental stage they are measured and in which species they are measured.

In this study, the Pearson's correlation between dendritic field size and maximal instantaneous firing rate was 0.050 (p = 0.608) (Table 3), which indicated large dendritic field alpha RGCs and small dendritic field beta RGCs were not different in their firing rates. Therefore, the strong correlation between morphology and intrinsic excitability in cat RGCs did not exist in developing mouse RGCs. In monkey, the receptive field of ON parasol RGC is 20% larger than that of the OFF parasol RGC, suggesting a larger dendritic field in ON RGCs [37]. In developing mouse, the ON, OFF and multistratified RGCs did not differ in morphology except their dendrites stratified in different sublaminas of the IPL and multistratified RGCs had longer total dendrite length and higher dendrite density. However, as RGCs mature, their morphology and intrinsic excitability change. Comparing to RGCs that fired action potentials repetitively, RGCs that only fired a single action potential had smaller dendritic fields, shorter total dendrite lengths, fewer branches and shorter branch lengths. This indicates that the physiologically less mature RGCs were also morphologically less mature.

Many studies have statistically classified mouse RGCs based on either morphology or physiology, but they each had limitations. Mouse RGCs were divided into 14 classes by a hierarchical method using 15 morphological parameters [24] or 11 classes by K-means method using three morphological parameters [26]. Because the morphological parameters are not independent, the parameters that describe the size of the RGC and the length of the dendrites probably dominated in Coombs' classification [24]. Kong and colleagues [26] acknowledged the redundancy in their 26 parameters and used only three independent parameters, depth of stratification in the IPL, dendritic field area and dendritic density, for classification. This avoided redundancy but lost information contained in other parameters. In a physiological study, mouse RGCs formed clusters based on their response latency, response duration and relative amplitude of the ON and OFF responses, but formed a single continuous group based on the degree of nonlinearity in the stimulus-to-response transformation [11]. In another recent physiological study, RGCs clustered into 12 subtypes based on their relative amplitude of the ON and OFF responses, response latency, response transience, direction selectivity and the receptive field surround [14]. These recordings were done by multielectrode recording and did not reveal RGC morphology.

This study attempted to group RGCs based on both their morphological properties and intrinsic excitability. Twelve morphological parameters and seven physiological parameters were combined to search for RGC subtypes that were both morphologically and physiologically different from each other. However, as described above, these parameters varied greatly in magnitude, and many were highly related to one another. PCA was applied to extract independent factors out of the nineteen original parameters without the bias of presumptive selection and weighting. PCA yields independent factors fewer than the number of the original parameters and reduces the dimensions of the variables without losing the information contained in them. If there were distinct RGC subtypes, they would form clusters in the space defined by the orthogonal principal components. Such clusters did not form for mouse RGCs. Therefore, although mouse RGCs vary in morphology and physiology, the differences in morphology were not strongly related to differences in intrinsic excitability in postnatal RGCs. The variation in intrinsic excitability across morphological subtypes was more likely to be a continuous change instead of distinct clusters.

### The limitations and challenges

It should be noted that in this study the lack of correlation between morphology and physiology may be due to development that continues into early adulthood as visual experience is accumulated. This study only investigated the morphology and physiology in the early postnatal weeks. As the RGCs mature further, correlations shall emerge between morphology and intrinsic excitability [29], but perhaps like in rats, not to the extent that there are unique types in cat and other carnivores [20]. However, it is interesting to find out the lack of difference around eye opening, when RGCs have already acquired relative maturity of both morphology [21], [22] and physiology [38].

In addition, this study did not analyze RGC light responses. RGC light response is determined by both intrinsic excitability and synaptic input. The synaptic connection of RGCs to bipolar and amacrine cells is further determined by both the lateral branching pattern and the z-level of stratification of RGC dendrites in the IPL. Level of stratification in the IPL was found very important among the 42 morphological parameters examined by Kong and colleagues [26]. The RGCs in our study were comprehensively analyzed for their lateral branching patterns and were clearly identified to stratify in the ON and/or OFF sublaminas in the IPL. However, limited z-resolution in live tissue under epifluorescence microscope restrained further more sophisticated analysis on the z-level of stratification. Although intrinsic excitability was not strongly correlated to the morphology, different synaptic connections would still result in different RGC light responses [39].

It is extremely complicated to study the correlations between the morphology and physiology in mouse RGCs. The mouse RGC morphology has been analyzed quite comprehensively and it is generally agreed that there are about 15 morphological subtypes. However, the physiology of RGCs is much more complex. It can be grossly divided into intrinsic excitability and light responses. Depending on the stimuli, measuring methods and parameters measured, different classifications exist [11], [14]. To make things more complicated, RGCs change their physiological properties under different conditions [40]. In addition, the action potentials measured do not always reflect the underlying mechanisms [41]. Therefore, it requires a tremendous database to make a comprehensive analysis of the correlations between RGC morphology and physiology. There are multiple ways to achieve the same outcome of action potential patterns. Having large databases to search for combinations of morphological and physiological parameters that generate a common action potential patterns will help determine how RGCs generate specific action potential patterns and how stable they are over development and changes in ambient conditions. Without this data the argument of the degree of correlation that has been going on in this field for decades will continue without conclusive results. Although this study did not resolve this debate, it offers valuable insights on the properties measured and contributes to our overall understanding of whether and how RGCs have types with consistent morphology and physiology, suggesting that physiological differences among RGCs develop later than anticipated in mouse and with less strong correlations to morphology than in other species.

## Supporting Information

Table S1 **Number of recorded RGCs at different developmental ages.**
(DOC)

Table S2 **Six independent factors explaining 78% of the original parameters were extracted from the original parameters by PCA.**
(DOC)

Table S3 **The factor loading scores of the original parameters.** Each factor was a combination of all the original parameters. Factor 1 mainly described the size of dendritic field and the length of dendritic branches. Factor 2 was more related to total dendrite length and number of branches. Factor 3 represented dendrite diameter, action potential width and firing rate. Factor 4 was dominated by the resting membrane potential and the difference between resting potential and action potential threshold. Factor 5 was mainly symmetry and factor 6 was mainly tortuosity.
(DOC)
