# Supplementary material for: The Morphology and Intrinsic Excitability of Developing Mouse Retinal Ganglion Cells
Source: PLoS One. 2011 Jul 13;6(7):e21777. doi: 10.1371/journal.pone.0021777 (PMC3135603; doi:10.1371/journal.pone.0021777)
Supplement: Table S1 — Number of recorded RGCs at different developmental ages. (DOC) [file pone.0021777.s001.doc]

**Table S**1. Number of recorded RGCs at different developmental ages.

| Age (days postnatal) | RGCs recorded in Qu and Myhr 2008 [26] | Additional RGCs newly recorded in this study | Total RGCs |
| --- | --- | --- | --- |
| 4 | 8 | 10 | 18 |
| 5 | 9 | 1 | 10 |
| 6 | 7 | 3 | 10 |
| 8 | 3 | 0 | 3 |
| 9 | 19 | 9 | 28 |
| 10 | 16 | 7 | 23 |
| 11 | 8 | 9 | 17 |
| 12 | 8 | 10 | 18 |
| 13 | 7 | 6 | 13 |
| 14 | 8 | 2 | 10 |
| 16 | 0 | 4 | 4 |
| 20 | 8 | 3 | 11 |
| 21-24 | 6 | 4 | 10 |
| Total | 107 | 68 | 175 |
