# Supplementary material for: The Morphology and Intrinsic Excitability of Developing Mouse Retinal Ganglion Cells
Source: PLoS One. 2011 Jul 13;6(7):e21777. doi: 10.1371/journal.pone.0021777 (PMC3135603; doi:10.1371/journal.pone.0021777)
Supplement: Table S2 — Six independent factors explaining 78% of the original parameters were extracted from the original parameters by PCA. (DOC) [file pone.0021777.s002.doc]

**Table S2. Six independent factors explaining 78% of the original parameters were extracted from the original parameters by PCA.**

| Factors | Total Variance Explained | % of Variance | Cumulative % |
| --- | --- | --- | --- |
| 1 | 4.583 | 25.462 | 25.462 |
| 2 | 3.392 | 18.842 | 44.304 |
| 3 | 2.034 | 11.299 | 55.603 |
| 4 | 1.658 | 9.213 | 64.815 |
| 5 | 1.282 | 7.121 | 71.936 |
| 6 | 1.122 | 6.235 | 78.172 |
