# Supplementary material for: The Morphology and Intrinsic Excitability of Developing Mouse Retinal Ganglion Cells
Source: PLoS One. 2011 Jul 13;6(7):e21777. doi: 10.1371/journal.pone.0021777 (PMC3135603; doi:10.1371/journal.pone.0021777)
Supplement: Table S3 — The factor loading scores of the original parameters. Each factor was a combination of all the original parameters. Factor 1 mainly described the size of dendritic field and the length of dendritic branches. Factor 2 was more related to total dendrite length and number of branches. Factor 3 represented dendrite diameter, action potential width and firing rate. Factor 4 was dominated by the resting membrane potential and the difference between resting potential and action potential threshold. Factor 5 was mainly symmetry and factor 6 was mainly tortuosity. (DOC) [file pone.0021777.s003.doc]

**Table S**3. The factor loading scores of the original parameters.

| **Original Parameters** | **Factors** | | | | | |
| --- | --- | --- | --- | --- | --- | --- |
|  | 1 | 2 | 3 | 4 | 5 | 6 |
| Soma Area | .228 | .293 | .357 | -.255 | -.374 | .145 |
| Dendritic Field Area | .871 | .190 | .247 | -.032 | .074 | .001 |
| Total Dendrite Length | .518 | .696 | .198 | .131 | .248 | .045 |
| Dendrite Density | -.650 | .431 | -.155 | .226 | .280 | .079 |
| Number of Branches | -.269 | .847 | .316 | .036 | .148 | -.082 |
| Branch Order | -.530 | .480 | .408 | -.109 | .153 | .053 |
| Internal Branch Length | .868 | -.279 | -.163 | -.034 | .177 | .175 |
| Terminal Branch Length | .767 | -.395 | -.091 | .060 | .084 | .323 |
| Branch Angle | -.629 | .257 | -.398 | .180 | .010 | .031 |
| Dendrite Diameter | .185 | .188 | .530 | -.007 | -.194 | .290 |
| Tortuosity | -.197 | .213 | -.470 | .265 | .255 | .624 |
| Symmetry | -.029 | -.196 | .028 | -.218 | .703 | .205 |
| Cm | .607 | .581 | .264 | -.024 | .073 | -.166 |
| Rm | -.591 | -.437 | .173 | -.236 | -.123 | .130 |
| Vm | -.074 | -.510 | .309 | .708 | .268 | -.233 |
| APT | -.145 | -.517 | .076 | -.093 | .557 | -.445 |
| APT-Vm | -.032 | .169 | -.292 | -.884 | .140 | -.090 |
| AP Width | -.363 | -.395 | .576 | -.020 | -.104 | .260 |
| Firing Rate | .298 | .277 | -.575 | .271 | -.153 | -.283 |

Each factor was a combination of all the original parameters. Factor 1 mainly described the size of dendritic field and the length of dendritic branches. Factor 2 was more related to total dendrite length and number of branches. Factor 3 represented dendrite diameter, action potential width and firing rate. Factor 4 was dominated by the resting membrane potential and the difference between resting potential and action potential threshold. Factor 5 was mainly symmetry and factor 6 was mainly tortuosity.
